# Evaluation of the influence of the genetic background in tissue repair in diabetic mice

**DOI:** 10.1186/1758-5996-7-S1-A244

**Published:** 2015-11-11

**Authors:** Simone Aparecida de Almeida, Silvia Passos Andrade, Paula Peixo Campos Lopes, Laura Alejandra Ariza Orellano, Celso Tarso Rodrigues Viana, Luciana Xavier Pereira, Mônica Alves Neves Diniz Ferreira

**Affiliations:** 1 Universidade Federal de Minas Gerais, Belo Horizonte, Brazil

## Background

Studies with genetically different strains of mice showed different responses for diabetes, glucose levels and insulin levels. Several experimental models have shown that wound healing and angiogenesis are phenotypes dependent on the collection of genes present in an organism. However, we found no study that investigated the influence of genetic heterogeneity in the process of inner healing in diabetic animals.

## Objectives

Evaluate the influence of genetic background in the components of repair process (angiogenesis and inflammation) induced by synthetic matrix in mice with type I diabetes (Swiss, Balb/C and C57).

## Materials and methods

Angiogenesis and inflammation were assessed at 10 days after implantation in polyether-polyurethane sponge discs implanted subcutaneously in female Swiss, C57, and Balb/c control and diabetic mice induced by streptozotocin (STZ; n=10).

## Results

The strains responded distinctly to the diabetogenic treatment as assessed by fasting glucose levels (Swiss CT=134.0±3.8 vs STZ=455.4±14.51; C57 CT=135.4±6.2 vs STZ=393±21.7; Balb/c CT=118.4±4.0 vs STZ 190.0±10.46). Hemoglobin content, (µg/mg) in implants of Swiss diabetic animals decreased by 59% compared with the control group. The diabetogenic treatment did not alter this parameter in the other two strains. In all strains the number of vessels was decreased in implants of diabetic animals compared with their control groups (Swiss CT=47.5±14.8 vs STZ=6.5±3.5; C57 CT=47.5±6.2 vs STZ=5.0±1.9; Balb/c CT=39.5 ±11.4 vs BSTZ 10.0±3.5). In contrast, VEGF levels (pg/mg) were increased in implants of Swiss and C57/BL diabetic mice The inflammatory parameters (Myeloperoxidase-MPO; N-acetyl-B-D-glucosaminidase-NAG and NO) were markedly influenced by the genetic background. In implants of Swiss and Balb/c diabetic animals, MPO increased, but NAG activity was lower in implants of Swiss diabetic mice. Furthermore, the levels of nitric oxide were also reduced in implants of the all diabetic mouse strains. The inflammatory cytokines (TNF, CCl2 and KC) also showed distinct profiles after the diabetogenic treatment.

## Conclusion

The genetic background influenced the systemic and local response to the diabetogenic treatment in the strains of mice evaluated. Swiss mice were the most affected strain analyzed whereas the least was the Balb/c. These important strainrelated differences to diabetes must be considered in the design and analysis of studies internal healing hyperglicemic environment.

